# Gradient-Regulated Acid–Alcohol–Ester Metabolism in Stress-Tolerant Functional Units During Chinese Baijiu Solid-State Fermentation

**DOI:** 10.3390/foods15162843

**Published:** 2026-08-14

**Authors:** Yuting Zhang, Zheng Xu, Jian Zhang, Gaosen Zhang, Jie Cui, Yonghong Hu

**Affiliations:** 1 College of Biotechnology and Pharmaceutical Engineering, Nanjing Tech University, Nanjing 211816, China; 202321166118@njtech.edu.cn (Y.Z.); yonghonghuyg@163.com (Y.H.); 2 College of Food Science and Light Industry, Nanjing Tech University, Nanjing 211816, China; xuzheng@njtech.edu.cn (Z.X.); four-leaf-clover@njtech.edu.cn (J.Z.); 3 Northwest Institute of Eco-Environment and Resources, Chinese Academy of Sciences, Lanzhou 730000, China

**Keywords:** Chinese Baijiu, solid-state fermentation, stress-tolerant functional consortium, ester biosynthesis, microenvironmental gradient, functional maintenance, gradient solid-state bioreactor, online monitoring

## Abstract

Chinese Baijiu solid-state fermentation operates as a heterogeneous reaction bed in which heat, moisture, O_2_, acidity, ethanol, and mass-transfer conditions vary across space and time. Because aroma-related microorganisms respond differently to these local stresses, abundance data alone cannot identify which populations remain active or contribute to ester formation at a particular layer, stage, or batch. This review therefore focuses on stress-tolerant functional units that sustain acid–alcohol–ester conversion and argues for spatially resolved sampling combined with activity-, enzyme-, and metabolite-based evidence. As a conceptual verification platform, rather than a reported experimental apparatus or a replacement for traditional pits, a gradient solid-state bioreactor could reproduce selected temperature, O_2_, moisture, and interface gradients under controlled conditions. Coupling multipoint temperature, CO_2_/O_2_, moisture, and acidification-related signals with spectral fingerprints and volatile measurements, together with offline biological assays, could help define functional windows and improve the mechanistic reproducibility of Baijiu fermentation.

## 1. Introduction

Chinese Baijiu is a traditional distilled spirit produced mainly through solid-state grain fermentation [1,2,3]. During fermentation, cooked grains, Daqu (a brick-shaped starter that supplies enzymes and microorganisms), yeasts, bacteria, filamentous fungi, acids, ethanol, and local gases coexist in a porous bed [1,2,3,4]. Unlike a well-mixed liquid culture, this bed develops spatial and temporal gradients in temperature, moisture, O_2_ availability, acidity, ethanol, and substrate accessibility [5,6,7]. Recent spatiotemporal sampling of Baijiu stacking fermentation further identified oxygen-depleted, transitional, and enriched layers with distinct microbial succession dynamics [8]. Consequently, samples from the upper layer, pit interior, wall region, or grain–pit mud interface can represent different local reaction conditions. Microbial composition alone therefore cannot explain flavor formation.

Research on Baijiu has provided comprehensive support for the studies of flavor components and microbial activities. Previous studies have listed the main microbial groups, flavor substances, and fermentation characteristics of Baijiu [1,2,3]. Recently, a review focusing on yeasts further detailed the different metabolic characteristics, stress tolerance, and applications of brewing yeasts and non-brewing yeasts [9]. Other studies have also explored the regulation of microbial communities, functional strains, biological enhancement, and systems biology methods in the fermentation of Baijiu [10,11].

Esters are major contributors to Baijiu flavor [12,13,14]. Published studies separately document precursor supply, ester-related enzymes, and microbial interactions, but microbial abundance alone does not establish ester production [12,13,15,16,17,18,19,20,21,22,23]. A microorganism and a volatile compound may increase together because both tolerate the same local stress, occupy the same layer, or respond to a decline in competitors. Such co-occurrence is associative, not direct evidence of metabolic contribution.

This article only focuses on the stress-tolerant ester-producing functional units, rather than the entire microbial community of Baijiu. The functional units here refer to a group of microorganisms that can work together under stress to regulate the acid–alcohol–ester balance in the local process. Members may include ester-producing yeasts such as *Saccharomyces cerevisiae*, lactic acid bacteria, *Bacillus*, filamentous fungi, and anaerobic acid producers [15,16,17,18,24,25,26,27,28,29,30,31]. Not all member identities are based on taxonomy. Only when the activity, enzymatic capacity, or metabolic contribution of a microorganism can be linked to location, stage, physicochemical conditions, and ester outcomes should it be considered as part of the functional unit. Recently, co-culture experiments have shown that *Wickerhamomyces anomalus* and *Saccharomyces cerevisiae* can increase ester-related production through metabolic interactions [15,16,17]. Studies on biofilms and *Bacillus*–yeast co-cultures also indicate that changes in spatial state and microbial interactions can alter ester synthesis in strong-flavor Baijiu, a Baijiu type characterized by pit fermentation and ethyl hexanoate-rich aroma [18]. These results support the perspective of functional units, but they have not yet been studied in the context of a real solid-state gradient.

Therefore, stress tolerance is not an accessory trait. It may indicate that a strain or a microbial combination remains suitable for use even after the fermentation bed becomes acidic, alcoholic, hot, O_2_-limited, or moisture-limited. *Schizosaccharomyces pombe* has been proven to be able to tolerate 10 g/L acetic acid and reduce the acetic acid content in Zaopei (the fermented grain mixture in the fermentation pit) from 9.62 g/kg to 6.55 g/kg [32]. The above evidence links stress, metabolism and process functions. Metagenomic studies have also found that there are more carbohydrate-active enzymes in pit mud samples. In the metagenome analysis related to Daqu, glycosyltransferases and glycoside hydrolases were identified among the carbohydrate-active enzyme annotations [33]. This indicates that substrate release and precursor supply should be considered together with ester formation.

An engineering verification tool is therefore needed. Traditional pits preserve the production ecology but do not permit independent control of their gradients. Liquid cultures and simple simulated fermentations are reproducible, yet they do not reproduce the heat transfer, O_2_ limitation, moisture distribution, or diffusion resistance of a solid grain bed [5,7,34,35,36]. On the basis of established packed-bed and multi-layer solid-state fermentation (SSF) reactor principles [34,35,37,38,39], a gradient solid-state bioreactor is proposed here solely as a conceptual intermediate platform. It would neither replace traditional pits nor present unpublished performance data; instead, it would reproduce selected Zaopei gradients under controlled conditions to test whether a given functional unit retains its esterification capacity.

Monitoring should follow the same logic. The realistic route is not to install every advanced sensor, but to build a layered evidence system. Fast variables should include multipoint temperature, moisture, CO_2_ and O_2_, pH or acidification-related signals, near-infrared spectroscopy (NIR), Raman spectroscopy, and volatile organic compounds (VOCs) [40,41]. These variables can capture heat generation, gas metabolism, acidification, moisture migration and volatile changes at high frequency. They should then be combined with slower evidence, including quantitative polymerase chain reaction (qPCR), enzyme activity assays, gas chromatography–mass spectrometry (GC-MS), metatranscriptomics and targeted metabolite analysis. The purpose is not to replace offline confirmation, but to connect online process state with biological function.

Machine learning will be used after the identification of the biological target above. Recent studies that have used multi-dimensional sensor data and interpretable models for Baijiu fermentation-stage recognition have achieved a 99.48% accuracy with a multi-layer perceptron model, and Shapley additive explanations (SHAP) analysis has identified CO_2_, pH, temperature and moisture as the important predictors [42]. Although the above results provide support for the value of process signals, they should not be taken as proof that machine learning can perform Baijiu fermentation control independently. This paper reviews the following research in current studies and discusses why the stress-tolerant ester-producing function can still be displayed in microenvironmental gradients. Therefore, interpretable models, soft sensors and digital twin concepts should be used to connect fast online variables with slower biological and chemical data.

Thus, the central argument of this review is that the control target of Baijiu solid-state fermentation should move from microbial richness or volatile peak changes to the maintenance and verification of stress-tolerant ester-producing function. The following sections discuss the gradients that select functional units, the evidence needed to distinguish active function from co-occurrence, and the possible use of gradient solid-state bioreactors and online monitoring to make this question experimentally testable for Baijiu production.

## 2. From Traditional Process Units to a Gradient-Defined Functional System

### 2.1. Traditional Process Units as Sources of Microenvironmental Gradients

Chinese Baijiu fermentation is usually described through Daqu, Zaopei, pit mud, Huangshui (the yellowish acidic liquid that accumulates at the bottom of the pit), and distillers’ grains. The above description is applicable to process identification, but it does not help in understanding functional stability. From a scientific process perspective, these units can be further understood as the sources, carriers, and boundaries of gradients. The Daqu provides microorganisms, enzymes, and flavor precursors, and the functions of these substances are influenced by the temperature-adaptive microbial succession and enzyme formation during the brewing process [4,43,44,45,46,47,48,49]. The Zaopei is the main reaction bed, but it is not a homogeneous substrate. During the fermentation process, its upper, middle, and lower parts may have different temperatures, moisture contents, acids, ethanol, and reducing sugars, and the microbial communities will also be different [6,50,51,52,53,54,55]. The pit mud is a persistent anaerobic area that generates functional anaerobic bacteria and is a source of soluble metabolites [56,57]. Huangshui contains water, ethanol, acids, sugars, amino acids and microorganisms, and can be regarded as a liquid feedback phase rather than only a by-product [58,59].

**Figure 1:**
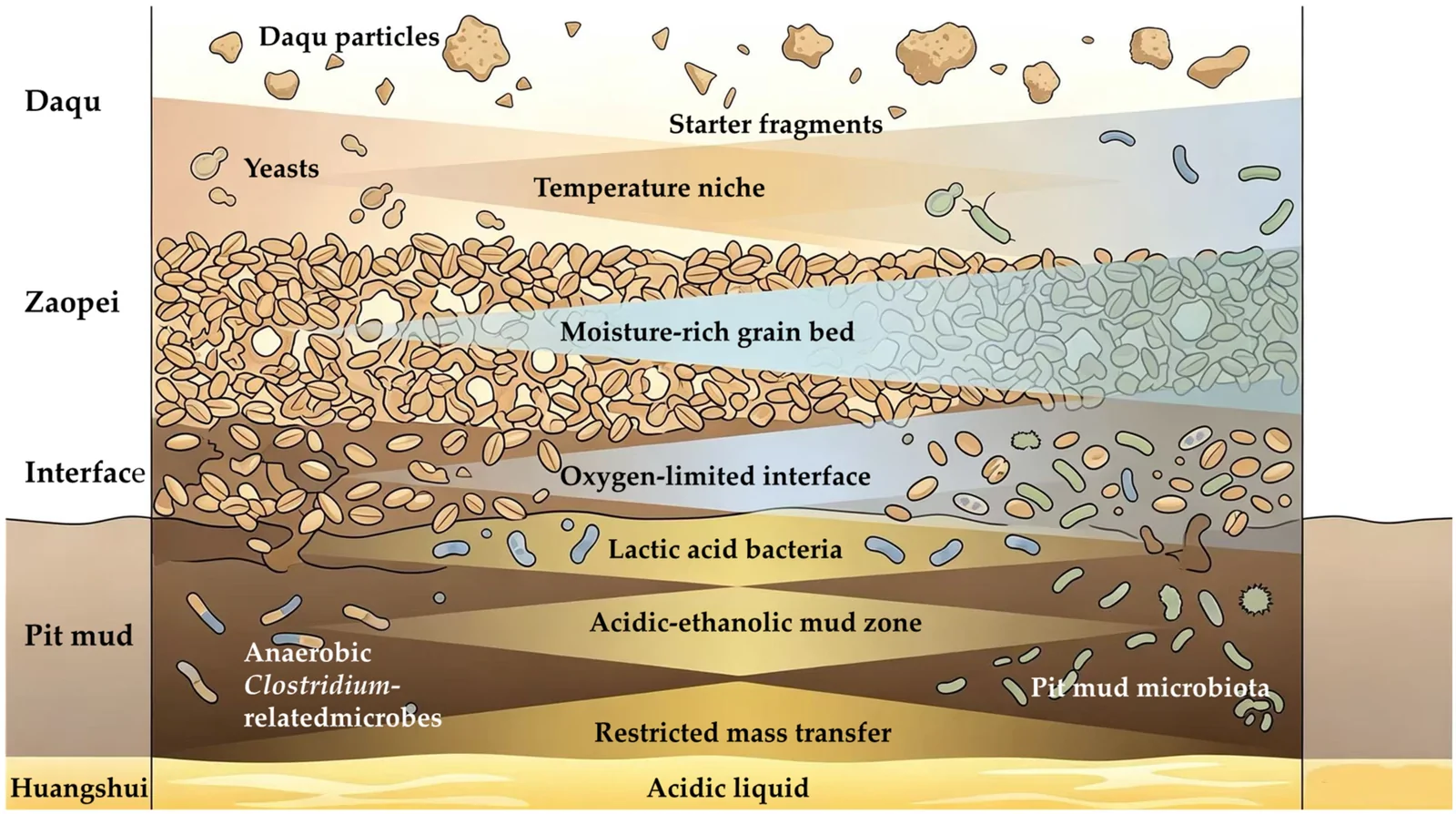
Spatial gradients and functional selection in traditional Baijiu solid-state fermentation. The schematic illustrates the spatial organization of Daqu particles, Zaopei, the grain–pit mud interface, pit mud and Huangshui in a traditional fermentation pit. Temperature, moisture, oxygen/redox state, acidity, ethanol accumulation and mass transfer form heterogeneous microenvironmental gradients, which selectively shape the survival, activity and metabolic contribution of yeasts, lactic acid bacteria, anaerobic bacteria and pit-mud microbiota.

Therefore, the key issue is not only which microorganisms are present in each unit, but also whether the microorganisms remain active under the local gradients where ester precursors and products are formed. As shown in Figure 1, the traditional pit can be understood as a gradient-defined reaction system in which Daqu particles, Zaopei, the grain–pit mud interface, pit mud and Huangshui form different microhabitats. These microhabitats impose selective pressures on microbial survival, enzyme activity, and metabolite transfer [6,7,56,57,58,59,60,61,62,63].

The process parameters in Table 1 are candidate descriptors for testing whether a functional unit remains active at a particular layer, time, or batch. Baijiu studies link temperature, moisture, acidity, and ethanol with community succession and flavor-related changes [6,13,50,51,52,53,54,55,60,61,62]; oxygen-resolved stacking data support spatial oxygen differentiation [8]; and pit-mud and Huangshui studies support interface-associated anaerobic metabolism and soluble-metabolite exchange [56,57,58,59,63]. Packed-bed studies further show that heat, water, and gas transfer depend on reactor geometry and operation [34,35,37,38,39]. These descriptors therefore connect published observations with testable engineering variables.

### 2.2. Microenvironmental Gradients Rather than Unit Names Determine Function

The same process unit can be in different functional states. For example, Zaopei near the pit wall or bottom is in closer contact with pit mud and Huangshui, and upper Zaopei may be exposed to different O_2_ levels, heat dissipation and moisture loss. Previous studies on strong-flavor (Luzhou-flavor) fermented grains have shown that there are different bacterial communities and biochemical characteristics in different layers [6,51,52,53,60]. Research on old and new Zaopei has shown that the condition of pit mud can change microbial succession, substrate consumption, ethanol production, acidity development and ester accumulation [50,55,61,62]. Based on the above results, Zaopei should not be regarded as a single biological unit in the discussion of microbial functions.

Water alters the availability of substrates, changes the diffusion rate of solutes and gases, affects enzyme activity, and thus stresses microbes in solid-state fermentation [7]. The initial moisture content of Baijiu fermentation is thought to be related to changes in the microbial community structure and flavor development [6]. Tolerance needs to be considered in a particular environment for the present review. A group of yeasts or bacteria that produce ester precursors in a mild environment will lose their activity if the water activity drops, the acidity rises, or ethanol is produced. Therefore, the functional units mentioned in this review need to be presented along with their operating parameters.

### 2.3. Interface-Driven Transfer Between Zaopei, Pit Mud and Huangshui

The interface between grains and cellar mud is a relatively unstable area. The cellar mud, as the container of the cellar walls, constitutes an anaerobic ecosystem. Research on artificial pit mud and long-term pit mud indicates that pit mud communities change with production conditions and can contribute to anaerobic metabolism [56,57,63]. Based on these results, this interface can be regarded as an active response area rather than a passive boundary.

Huangshui will also be included in this gradient system. It collects at the bottom of the pit and contains water, alcohol, acid, sugar, amino acid and microorganisms [58]. Huangshui is also an industrial by-product of pit fermentation. It indicates the decrease in soluble substances and may be involved in negative feedback regulation of acid concentration, anaerobic metabolism and precursor availability. Recently, research has been conducted on enrichment systems involving pit mud, which also shows that boundary materials can alter the structure of the microbial community and organic acid metabolism [59]. Huangshui does not need to be used directly without control; instead, a liquid-phase feedback mechanism may be required to build a gradient verification system.

### 2.4. From Abundance Evidence to Functional Maintenance Evidence

DNA-based community profiling identifies taxa but cannot reliably distinguish live, dead, or metabolically inactive cells. In Baijiu Zaopei, propidium monoazide (PMA) pretreatment has been combined with qPCR and amplicon sequencing to enrich signals from living microorganisms [70]. Metatranscriptomics has likewise been used to identify active microorganisms in light-flavor Baijiu fermentation [71,72]. These activity-sensitive approaches are necessary to determine whether stress-tolerant ester-producing units remain functionally active.

Therefore, the links in the chain of evidence should be four or more. The first level is for space-resolved physicochemical data, including temperature, relative humidity, pH, and ethanol and oxygen concentration. The second level is activity-sensitive microbial evidence, such as viable-cell analysis, qPCR and transcript- or protein-level information when available. The third stage is the enzymatic evidence of saccharification, acid generation, alcohol production and ester synthesis. The fourth level is the evidence of the target metabolites, such as alcohols, organic acids and essential esters. Only when the above levels are linked can a group of microbes be considered functionally maintained rather than just observed [72,73].

### 2.5. Engineering Implication: The Need for a Gradient Verification System

The traditional pit-type system is a good industrial setup, but it is not suitable for a controllable experimental platform. Liquid culture and simple simulation fermentation can enhance the reproducibility of experiments, but they cannot obtain the gradient structure found in solid-state fermentation systems. Therefore, this method has a drawback: the industrial field requires a system that is more feasible than the traditional open-pit system, but is more representative than test tubes or liquid co-culture models [5,7,34,35,36,37,38,39].

Packed-bed solid-state bioreactors have been used to examine heat transfer, O_2_ transfer, moisture distribution, and within-bed heterogeneity [34,35,36,37,38,39]. However, owing to the complexity of the Zaopei microbiota, the granular nature of the substrate, limited mixing, combined acetic acid–ethanol stress, and the presence of anaerobic zones, these systems cannot be directly transplanted to Baijiu fermentation. A Baijiu-oriented gradient platform should therefore reproduce selected pit gradients rather than optimize bulk microbial growth. In this way, the reactor concept can be used to determine whether the activity of a selected stress-tolerant esterifying functional unit is maintained under defined conditions of temperature, moisture, O_2_, acidity, and mass transfer.

## 3. Stress-Tolerant Ester-Producing Functional Units in Baijiu Fermentation

### 3.1. Functional Units Should Be Defined by Maintained Activity Rather than Taxonomic Presence

Section 2 states that the fermentation of Baijiu can be regarded as a gradient-defined solid-state system. Based on this viewpoint, the research object of this paper is not a single microorganism or the entire microbial community, but rather functional units with the ability to tolerate stress and produce esters. A group of microbial communities that can generate precursor substances and esters under specific stress conditions (such as low pH value, the presence of ethanol, temperature fluctuations, anaerobic environment, and reduced moisture transport) is called a community.

The definition above must have a clear and particular purpose; otherwise, it will be too general. The three conditions that the functional units must meet are as follows: they should be able to survive or remain active during the fermentation process; they need to be able to produce or regulate the precursor substances required for ester synthesis; and they should be able to promote the formation of the target esters in an observable way. Fermentation experiments have shown that simplified functional consortia and functional microbial combinations can improve key flavor compounds in Baijiu [15,16]. However, this combination should not be judged solely by the changes in the components of the final product. At the same time, a process environment close to that of the traditional solid-state system will be chosen for testing.

Esters are central to Baijiu flavor, but ester formation is not controlled by ester-producing microorganisms alone [12,13,14]. Ethanol-producing yeasts, organic acid-producing bacteria, saccharifying fungi, *Bacillus*-related enzyme producers and pit mud-derived anaerobes may all participate in precursor generation or conversion. Therefore, the term “ester-producing functional unit” in this review does not mean that every member directly synthesizes esters. It means that the unit as a whole maintains the acid–alcohol–ester chain.

### 3.2. Yeasts as the Main Alcohol- and Ester-Related Members

Yeasts are generally the first to be added to this function because they produce ethanol and many aroma-active substances. *Saccharomyces cerevisiae* is still frequently employed for its fermentability and tolerance to ethanol. A strain of *S. cerevisiae* YS219 has been shown to produce large amounts of ethyl hexanoate and is tolerant to organic acids; inoculation with this strain can alter the microbial community and flavor metabolite profile of strong-flavor Baijiu fermentation [24,25]. Therefore, *S. cerevisiae* should not be viewed solely as an alcohol producer, but under favorable community conditions, it may also help stabilize ester-related metabolism.

To determine whether some yeasts can serve as components in a stress-tolerant ester-producing unit. *Wickerhamomyces anomalus* has been used in simulated solid-state fermentation for Baijiu production, and changes in the microbial community structure and flavor metabolism have been observed [26,74,75,76]. Screening work for rice-flavor Baijiu also selected ester-producing yeasts with industrial stress tolerance, and it was determined that both ester production and tolerance should be considered in strain selection [77,78]. The above studies support a functional screening approach: a yeast candidate should not be selected solely based on its production of esters in a mild culture system, but rather due to the retention of aroma-related metabolism under fermentation-like stress.

*Pichia kudriavzevii* is also related; however, it should be mentioned in terms of tolerance and functional compatibility rather than as a general-purpose species. A heat-tolerant *P. kudriavzevii* strain with strong-flavor Daqu was isolated and, using transcriptomic and metabolomic analysis, its heat-response mechanism up to 50 °C has been elucidated [79,80]. Another study has identified a strain of acetic acid-tolerant *P. kudriavzevii* in Huangshui and found that it can tolerate as high as 12 g/L of acetic acid [81]. The above data provide a scientific basis for determining that *P. kudriavzevii* can be preserved in acidic or high-temperature stress in its microenvironment. However, its role needs to be confirmed in a mixed-fermentation system, as the production of acids, alcohols, esters, etc. may change due to the presence of other microorganisms. Table 2 shows the candidate microorganisms, their tolerance characteristics, functional contributions and evidence levels.

### 3.3. Lactic Acid Bacteria as Acidity Regulators and Interaction Partners

Lactic acid bacteria should not be known only as acid-producing microorganisms. They adjust pH during the production of Baijiu, add or modify various microbes, and supply substrates for ester production. Lactic acid can be turned into ethyl lactate, but under high-acidity conditions, ethanol-producing yeast are inhibited and the position of volatile compound emission changes. Lactic acid bacteria are thus necessary for acid–ester metabolism and, at the same time, generate a selective pressure.

Studies have shown that lactic acid bacteria and yeast isolated from light-flavor Baijiu interact to affect the fermentation metabolism of the Baijiu [27,28,29,30,31]. It can be assumed that lactic acid bacteria and yeast will be analyzed together rather than separately. Lactic acid bacteria in a functional unit can produce organic acids to regulate pH and inhibit yeast. The problem is not a deficiency of lactic acid bacteria; rather, the quantity of acid they produce is too low to promote esterification; thus, the reaction is hindered.

This is why the functional unit should also be connected to process monitoring. If the acidity rises and yeast activity and alcohol production decrease, then the cell is likely in an inhibitory acid-accumulation state. In the future validation of gradient bioreactors, lactic acid, acetic acid, ethanol and ester formation should all be measured concurrently rather than only total acid or total ester.

### 3.4. Bacillus, Filamentous Fungi and Ester-Related Enzymes

*Bacillus* and filamentous fungi are generally necessary to provide enzymes and precursor pathways. Daqu studies have shown that temperature-adapted microbiota can modify the profile of saccharification enzymes [19,20,33,86,87], and *Bacillus*-related functional microorganisms have been widely investigated for Daqu fortification and flavor production [73,88,89,90,91]. Based on the functional-unit framework, these microorganisms need to enhance precursor supply and maintain enzymatic activity in a solid-state gradient.

*Bacillus velezensis* is a typical case. Biofilm formation and co-culture with *B. velezensis* were found to affect ester production in strong-flavor Baijiu [18,91,92,93]. This is because biofilm or aggregated growth can increase the persistence of microbes in the event of stress in a porous grain matrix. However, there is also an engineering problem: whether to have such persistence without causing unstable community dominance. Therefore, *Bacillus*-related members should not be included in the functional unit merely because they are abundant in Daqu. They should be included only if their enzyme activity, interaction with yeasts or effect on ester precursors have been verified by experiment.

Filamentous fungi and *Monascus*-related enzymes are also worth studying. A short-chain fatty acid ester-synthesizing biocatalyst from *Monascus purpureus* was isolated in Baijiu and shown to have ester-synthesizing activity in an aqueous medium [94]. It does not directly show the same activity in the solid-state pit, but it provides a mechanism for how ester formation occurs at the enzyme level, rather than just microbial abundance and final volatile profiles. Therefore, enzyme assays and enzyme-source tracing can be used to connect microbial community analysis with flavor-compound detection.

### 3.5. Anaerobic Acid-Producing Members and Coupling with Yeasts

Strong-flavor Baijiu is produced by fermenting grains and pit mud together. Organic acid production is carried out by cooperation among microbial communities of fermented grains and pit mud [56,57,63]. It is necessary to have both ethanol and hexanoic acid-related precursors for the formation of ethyl hexanoate in the present article. Yeasts can produce ethanol, and anaerobic bacteria in pit mud may be responsible for generating short- and medium-chain fatty acids [28,95]. Therefore, the two will work together better than either can alone.

Recently, some targeted cooperation studies have taken place in this direction. Inoculation of *Clostridium tyrobutyricum* and *S. cerevisiae* in a simulated strong-flavor Baijiu fermentation system increased the production of key flavor metabolites [82]. A later co-culture experiment of *S. cerevisiae* and *Clostridium kluyveri* showed increased production and improved flavor stability of ethyl hexanoate [83]. These studies are not to provide an end-to-end industrial route, but rather to offer an experimental basis: ester formation can be enhanced by engineering metabolic cooperation between ethanol-producing and acid-producing organisms.

Direct inoculation of anaerobes into traditional pits remains technically challenging and requires careful validation. These functions are under the control of oxygen limitation, redox status, substrate availability and other interactions in the community. Therefore, anaerobic members should be included in the verified functional unit only after their activity has been confirmed under gradient conditions. It is also necessary to build a gradient solid-state bioreactor in the following parts [82,83,84].

### 3.6. Progress in Stress-Tolerant Functional Consortia

Recent studies on Baijiu functional microorganisms have moved from single-strain isolation to stress-tested microbial combinations. The useful evidence is no longer only whether a strain produces esters, but whether it maintains activity under acid, ethanol, heat, moisture and oxygen limitation. Reported examples show this shift. *Schizosaccharomyces pombe* tolerated 10 g/L acetic acid and decreased acetic acid in fermented grains from 9.62 to 6.55 g/kg [32]. A heat-tolerant *Pichia kudriavzevii* strain isolated from strong-flavor Daqu was studied under 50 °C stress [79], and another acetic acid-tolerant *P. kudriavzevii* strain from Huangshui tolerated 12 g/L acetic acid [81]. These values should be treated as screening thresholds rather than direct industrial control targets.

Co-culture studies provide another level of evidence. Yeast–yeast, yeast–*Bacillus*, and yeast–anaerobe combinations have been used to improve ester-related metabolism [29,30,82,83,96]. In pit-mud-oriented intervention, *Caproicibacterium lactatifermentans* reached 29.16% abundance in fortified pit mud and promoted fatty acid and ethyl ester formation during short-term use [84]. Fortified Daqu also showed layer-dependent effects: ethyl hexanoate increased by 15.9%, 15.5% and 27.2% in the top, middle and bottom layers, respectively, while ethyl acetate decreased by 5.4%, 11.1% and 26.6% [75,85,91]. These data indicate that functional regulation is possible, but the effect is position-dependent and should be verified under gradient conditions.

For this review, a functional unit should be evaluated by four quantitative parameters: stress survival range, precursor conversion, ester-related enzyme activity, and target ester trajectory. A strain that tolerates acid but causes acid accumulation is not sufficient. A strain that produces esters in liquid culture but loses activity in a low-moisture grain bed is also not sufficient. The useful unit is the smallest microbial combination that maintains acid–alcohol–ester conversion under defined stress.

### 3.7. Criteria for Selecting a Stress-Tolerant Ester-Producing Unit

Selection of a stress-tolerant ester-producing unit should follow four steps. First, candidate strains or combinations should be screened under acid, ethanol, temperature, and moisture stress. Second, the production and consumption of ethanol, lactic acid, acetic acid, butyric acid, and hexanoic acid should be quantified. Third, target esters should be measured in a solid-state matrix rather than only in liquid culture. Finally, the unit should be tested across spatially defined gradients to determine where its function is maintained or lost.

This approach avoids two common problems. The first problem is overinterpreting microbial abundance. The second problem is selecting strains based only on single-function assays. A strain with high ester yield in liquid culture may fail in a low-moisture grain bed. A strain with high acid tolerance may overproduce acids and inhibit yeast metabolism. A functional unit is therefore not the strongest single strain, but the smallest stable combination that maintains the acid–alcohol–ester chain under defined stress.

## 4. Acid–Alcohol–Ester Balance as the Functional Output

### 4.1. Ester Formation Depends on Precursor Balance

A stress-tolerant functional unit should be evaluated by its effect on the acid–alcohol–ester chain described in Section 3. Baijiu esters are formed through enzyme- or chemical-catalyzed reactions between alcohols and organic acids. Ethyl hexanoate, ethyl acetate, ethyl lactate, and ethyl butyrate are major contributors to Baijiu aroma [12,13,14,97]. The control objective is therefore not maximal total ester concentration, but a stable and interpretable balance among precursor acids, alcohols, and ester products.

Ethanol is mainly produced by yeasts. Lactic acid bacteria, acetic acid bacteria, *Bacillus*-related organisms and anaerobic acid-producing bacteria all produce organic acids. The supply of substrates, enzyme activity, moisture content and local pH also restrict the production of esters. Therefore, the function of this module will be to maintain the flow of the precursor, not to create a single-compound module that can operate independently.

**Figure 2:**
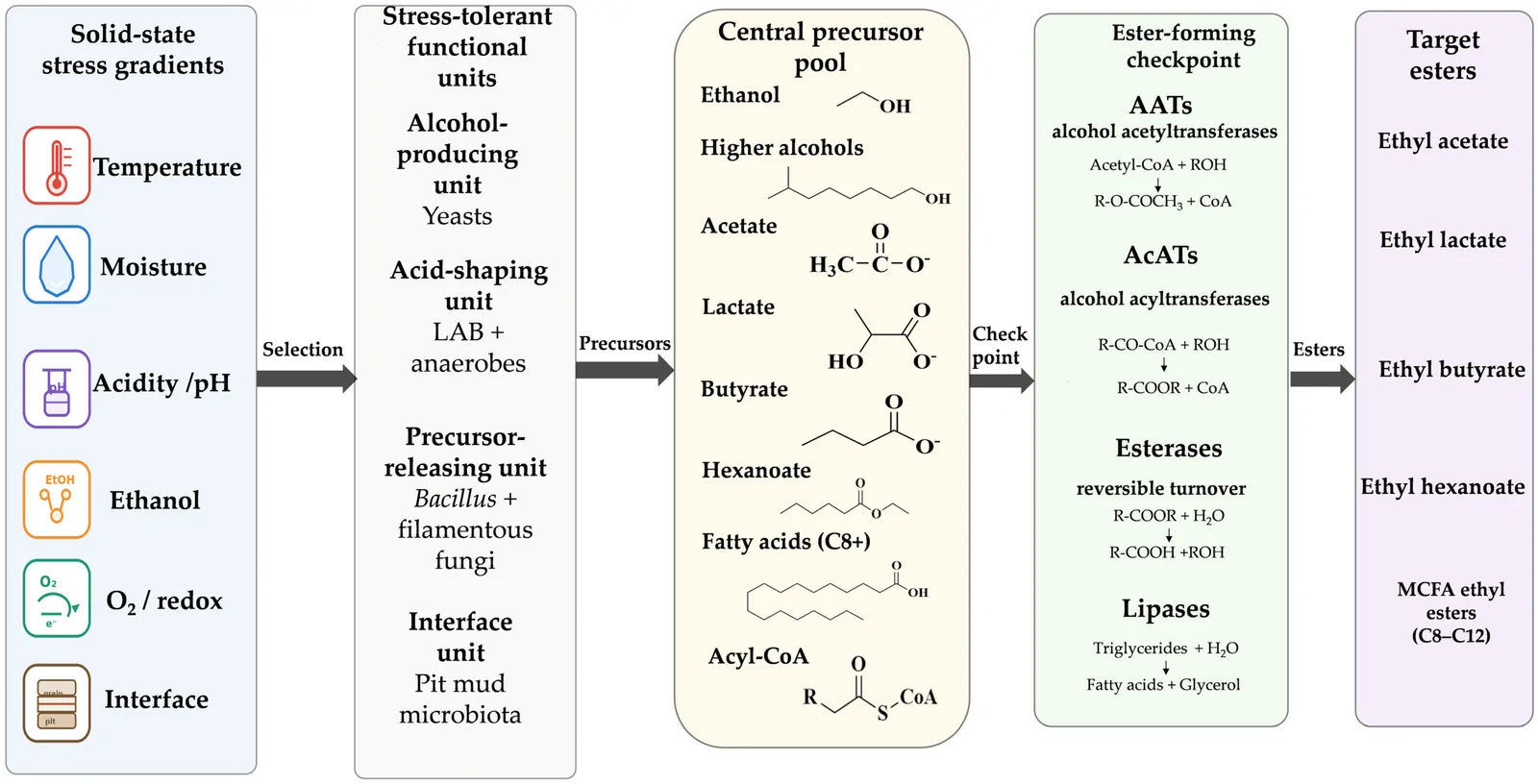
Stress-gradient-mediated acid–alcohol–ester conversion by functional microbial units. The schematic links solid-state stress gradients with stress-tolerant microbial units, central precursor pools, ester-forming enzymatic checkpoints and target ester outputs.

Ester formation in Baijiu should therefore be interpreted as a continuous functional chain rather than as the endpoint of a single microbial pathway. As summarized in Figure 2, local solid-state gradients first determine which microbial members can remain active under heat, moisture limitation, acidity, ethanol accumulation, and O_2_/redox stress [6,7,8,36]. The selected functional units then regulate the availability of central precursors, including ethanol, higher alcohols, acetate, lactate, butyrate, hexanoate, fatty acids and acyl-CoA. Whether these precursors are converted into aroma-active ethyl esters depends on alcohol acetyltransferases, alcohol acyltransferases, esterases, and lipases [19,20,21,22,23,74]. In this framework, ethyl acetate, ethyl lactate, ethyl butyrate, ethyl hexanoate and medium-chain fatty acid ethyl esters are not treated as isolated products, but as functional readouts of whether the acid–alcohol–ester chain is maintained under solid-state stress.

### 4.2. Ethanol Supply Is Necessary but Not Sufficient

Yeasts provide ethanol for ester synthesis, but ethanol supply alone cannot ensure ester accumulation. Ethyl hexanoate formation requires ethanol and hexanoic acid or hexanoyl-CoA as direct precursors. Metabolic engineering of *S. cerevisiae* showed that introduction of the hexanoyl-CoA pathway and alcohol acyltransferase genes increased ethyl hexanoate production, indicating that precursor activation and transferase activity affect final ester formation [22,74]. This supports the view that ethanol-producing yeasts need to be coupled with acid-producing or acyl-CoA-forming members.

Therefore, in the production of industrial Baijiu, only the amount of ethanol produced by yeast activity is not monitored. A stable functional unit should be able to produce ethanol and not inhibit the acid-producing partner. If the yeast grows too rapidly, there will be a relatively high demand for oxygen and substrates, as well as acid production. If yeast activity is inhibited by acid or ethanol stress, organic acids will accumulate and cannot be converted into esters. Both states will reduce process instability.

### 4.3. Organic Acid Accumulation Has a Threshold Effect

Organic acids are ester precursors; otherwise, they will accumulate to inhibit microbial activity or alter flavor. Lactic acid is needed to produce ethyl lactate, and acetic acid is related to ethyl acetate. Hexanoic acid and butyric acid are related to ethyl hexanoate and ethyl butyrate. However, these acids also change the pH and cause membrane damage and community succession. Acid metabolism is, therefore, considered to be in a regulated intermediate state rather than an independent positive factor.

The fermented grain–pit mud interface is an important site of organic acid formation. Cooperation among the two microbial consortia has been shown to promote the production of organic acids in strong-flavor Baijiu [13,31,56,57,63]. Hexanoic acid-producing bacteria can use ethanol, acetic acid, glucose, or lactic acid as carbon sources and provide hexanoic acid for ethyl hexanoate synthesis [28,95]. The above studies have shown that acid formation occurs at various locations and by different microbes. Therefore, the function of a module can be judged by its structure of acid generation and ethanol supply.

### 4.4. Esterification Should Be Linked to Enzyme Evidence

Ester synthesis in Baijiu is often discussed through final volatile compounds, but enzyme-level evidence is needed to support causality. Ethyl ester synthesis can involve esterase-catalyzed esterification and alcohol acyltransferase-related pathways with acyl-CoA intermediates [19,20,21,22,23]. Daqu provides both microorganisms and enzymes, and aqueous-phase ester synthase activity has been investigated as a possible contributor to ester formation [21]. This is important because Baijiu fermentation is not a dry chemical system; enzyme activity occurs in local aqueous phases within the grain bed.

Therefore, the extent of esterification should be indicated by the three linked indicators listed below: precursor acids, ethanol and ester-forming enzyme activity. Only measuring the final ester content will not help us determine whether the cause is an increase in precursor supply, an increase in enzyme activity, a decrease in hydrolysis or changes in volatility during distillation. Therefore, the enzyme assay falls between microbial evidence and volatile compound evidence in the proposed framework.

### 4.5. Functional Maintenance Should Be Judged by Ratios and Trajectories

The practical target is not maximal ester formation, but a stable acid–alcohol–ester trajectory. Studies on pits, Zaopei and Baijiu flavor chemistry showed that volatile compounds, acids and related microorganisms change during fermentation, and that ethyl acetate, ethyl hexanoate, ethyl lactate and ethyl butyrate are associated with different microbial groups and pit conditions [12,50,55,57,61,98]. This supports the use of dynamic ratios rather than single endpoint concentrations [99,100].

Three indicators of the evaluation of the process are more informative than the total ester alone: the acid-to-alcohol ratio, the conversion relationship between acid precursors and their corresponding ethyl esters, and the time-course trend of main ester ratios. The above indices will indicate whether the functional module is performing conversion or merely generating intermediates. Therefore, in the future gradient reactor experiments, sampling should be performed on different layers and stages, not just the final distilled liquor.

## 5. Gradient Solid-State Bioreactor as a Verification Platform

### 5.1. Rationale and Literature Basis for the Conceptual Platform

Traditional pits reproduce authentic fermentation ecology, but published studies show that physicochemical conditions, community succession and metabolites vary together across time, position and boundary materials [6,50,51,52,53,54,55,56,57,58,59,60,61,62,63]. These observational data cannot by themselves assign a change in a target ester uniquely to microbial activity, precursor supply, enzyme activity or mass transfer.

Flask cultures and small simulated fermentations improve reproducibility but omit important solid-state gradients [5,7,34,35,36]. This review therefore proposes a gradient solid-state bioreactor as a conceptual intermediate between laboratory culture and traditional pits. The platform is not presented as a fabricated apparatus, a new dataset or a replacement for pit fermentation; its intended use is to test whether selected functional units maintain acid–alcohol–ester conversion across controlled gradients.

**Figure 3:**
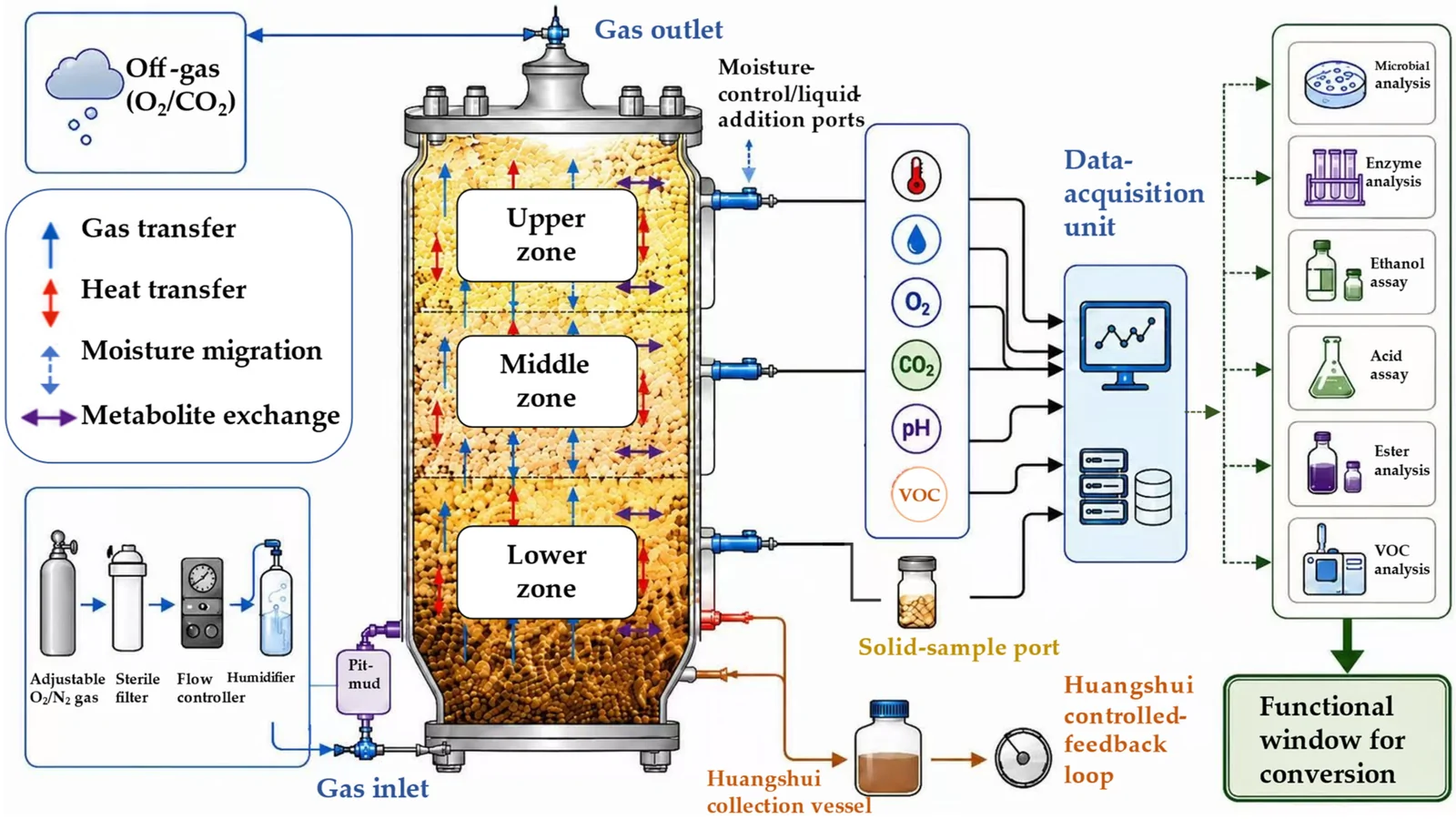
Conceptual gradient solid-state bioreactor for functional verification. The schematic shows the gas inlet and outlet, layered bed, liquid-addition and solid-sampling ports, multipoint sensors, interface-feedback modules, data acquisition, and offline endpoints used to define the functional window for acid–alcohol–ester conversion.

Figure 3 presents an original conceptual synthesis based on established packed-bed and multi-layer solid-state fermentation reactor designs [34,35,37,38,39] and on reported Baijiu gradients and interface effects [6,7,8,50,51,52,53,54,55,56,57,58,59,60,61,62,63]. The scheme identifies the variables that a future verification platform should reproduce—bed height, temperature distribution, moisture migration, O_2_ limitation, acidity, ethanol accumulation, pit-mud/interface contact and liquid-phase feedback—and couples these engineering variables to online monitoring and position-matched biological, enzymatic and metabolic endpoints.

### 5.2. Progress from Solid-State Reactors to Baijiu-Oriented Gradient Reactors

Solid-state fermentation reactors developed in other fields provide useful engineering references for Baijiu, but they solve different problems. Tray reactors improve operation simplicity but have weak control of internal gradients. Packed-bed reactors maintain a static bed and allow gas-flow control, making them useful for studying oxygen transfer, CO_2_ release, heat accumulation and bed-scale heterogeneity [34,35,37,38,39]. Zymotis-type reactors use internal heat-transfer plates to reduce overheating in static beds [34]. Multi-layer packed-bed reactors reduce heat accumulation by dividing the substrate into layers. Rotating-drum or intermittently mixed reactors improve homogeneity, but they may damage the spatial structure required for Baijiu-like fermentation.

For Baijiu, the design target is different. The reactor should not eliminate gradients. It should reproduce selected gradients in a measurable way. Therefore, a Baijiu-oriented gradient reactor should include at least four modules: a layered Zaopei bed, a controllable temperature boundary, an oxygen or gas-transfer boundary, and an exchange module representing pit mud or Huangshui contact. The realistic route is not to replace traditional pits with one vessel, but to combine traditional fermentation, insertable monitoring modules and locally engineered reactors for screening, comparison and data learning.

### 5.3. Engineering Parameters That Should Be Reported

A gradient reactor should be described by engineering variables, not only by the name of the reactor. The minimum parameters should include bed height, layer thickness, substrate loading density, initial moisture, boundary temperature, temperature-control accuracy, gas-flow direction, inlet and outlet O_2_/CO_2_, pressure drop, sampling-port depth, pit-mud contact area, Huangshui feedback volume and sampling interval [37,38,39]. These parameters determine whether the system is comparable between experiments.

For functional-unit validation, the reactor should also record biological and chemical endpoints at the same positions. The minimum endpoint set should include viable target microorganisms, qPCR abundance or transcript signal, saccharification activity, esterification-related activity, ethanol, lactic acid, acetic acid, butyric acid, hexanoic acid, ethyl acetate, ethyl lactate, ethyl butyrate and ethyl hexanoate. If these data are not position-matched, the reactor only provides another simulated fermentation system and cannot prove functional maintenance.

### 5.4. Quantitative Design Logic

The reactor should be built around measurable ranges. Temperature should be recorded at multiple depths because heat accumulation is the most direct scale-up risk in solid beds. Moisture should be measured by layer because water controls enzyme diffusion and substrate accessibility. Gas data should include O_2_ and CO_2_ because they distinguish aerobic, microaerobic and anaerobic zones. The reactor should not be judged by whether it produces more total ester, but by whether it can define the failure boundary of the selected functional unit.

A useful output is a functional window. For example, a unit can be described as active only within a defined temperature range, moisture range, acidity range and oxygen condition. Once ethanol formation declines, acids accumulate without ester conversion, or viable target cells decrease, the boundary of the unit has been reached. This changes the reactor from a production model into a verification platform. Different solid-state fermentation systems vary in their ability to preserve or control spatial gradients. Table 3 compares traditional pits, small-scale solid-state models and representative reactor configurations, highlighting why packed-bed and multi-layer systems are more suitable for Baijiu-oriented gradient verification than fully homogenized systems.

### 5.5. Verification Endpoints Should Be Linked to Function

A reactor will record temperature and gas data. Its output must be related to functional maintenance. A minimum of four endpoint groups need to be provided.

First of all, the end products need to have a wide range of temperatures, moisture contents, pH or acidity values, oxygen levels, etc. Second, microbial endpoints should be viable or active populations of the selected members. Thirdly, the enzymatic endpoints should include saccharification-related activity and esterification-related activity. Fourth, the metabolic endpoints should include ethanol, lactic acid, acetic acid, butyric acid, hexanoic acid, ethyl acetate, ethyl lactate, ethyl butyrate and ethyl hexanoate.

The above endpoints should be measured at the same time to determine the gradient at which the functional unit reaches acid–alcohol–ester conversion in the reactor. It is more practical to show that a strain increases only one ester in a flask or a simulated fermentation. It can also serve as a testing platform for the functional starter and Daqu fortification strategy or pit mud interface materials prior to industrial trials.

## 6. Monitoring and Data Fusion for Functional Maintenance

### 6.1. Monitoring Should Answer a Defined Biological Question

Monitoring in this review does not refer to the general fermentation process. It will determine whether the chosen functional group keeps the acid–alcohol–ester chain in a gradient. Therefore, the monitoring variables will be divided into two categories: fast-process signals and slow-validation evidence.

Fast signals include temperature, moisture, O_2_, CO_2_, acidity-related signals, NIR spectra and VOC fingerprints. These signals can indicate process deviation but cannot alone prove microbial function. Slow evidence includes viable-cell analysis, qPCR, enzyme activity and targeted metabolites. These methods are less real-time but are needed to confirm whether the functional unit remains active.

A model that predicts the fermentation stage is convenient, but it does not represent the underlying mechanism. Based on the above findings, we investigate whether the monitoring system has deteriorated and thus reduced the production of ethanol, increased acid generation, or decreased ester conversion rates.

### 6.2. Gas and Moisture Signals Indicate Metabolic State

CO_2_ and O_2_ are generally employed to measure the extent of respiration and fermentation and gas transfer. Packed-bed SSF studies use gas transfer and oxygen-uptake information to evaluate physiological status, heat removal and scale-up behavior [38,39]. The above methods are suitable for this purpose because they are non-destructive and can be connected to the reactor.

Altered moisture distribution restricts enzyme diffusion and limits substrate supply for microorganisms. Studies on water in solid-state fermentation and SSF reactor design show that moisture and water activity are directly related to diffusion, heat transfer and microbial activity [7,36]. The same idea can be used for multi-layer moisture tracking of Baijiu. We measure the water content of the substrate and determine whether the local moisture conditions are suitable for the chosen functional unit.

### 6.3. Spectral and Volatile Signals Provide Rapid Process Fingerprints

NIR can be used to determine the quantity of water, starch, acid and other chemical characteristics of fermented grains. NIR systems have been used for state-variable determination and process monitoring in solid-state fermentation [40,101]. NIR is suitable for fast layer screening in the above system and can be periodically calibrated in a lab.

Another type of evidence is that from Raman-based methods. Single-cell Raman spectroscopy has been proposed for ecology–microbiome engineering in Baijiu fermentation [102]. This method is not yet a general-purpose industrial instrument, but it can be used to determine whether microbial cells have metabolic activity under stress. It can conduct strain or community screening before reactor-scale experiments.

Volatile detection should focus on VOC trajectories. Rapid volatile fingerprinting has been used for Baijiu aroma and regional-origin assessment [41,100]. Electronic nose systems can also respond to volatile compounds in alcoholic beverages, but their signals require calibration against chemical data [103,104,105]. Therefore, VOC sensors should be used as early-warning or classification tools, while GC-MS, gas chromatography–ion mobility spectrometry (GC-IMS), or targeted chromatography remains necessary for compound-level validation.

Monitoring methods should be compared by measurable performance rather than by technical novelty. The available evidence supports fast screening by NIR, volatile fingerprinting, e-nose and data-fusion methods, but these signals should be treated as indirect indicators [40,41,101,103]. Specific device ranges and response times should be reported from the selected instrument specifications in future experiments rather than generalized as biological evidence.

### 6.4. Offline Assays Are Needed as Anchors

Online signals need offline anchors. The functional unit of the proposed system will contain microorganisms, enzymes and metabolites. Viable-cell methods and qPCR can determine whether the target genes are still expressed. Enzyme assays are used to measure the retained saccharification- and esterification-activities. Targeted metabolite analysis should be used to quantify ethanol, lactic acid, acetic acid, butyric acid, hexanoic acid and their corresponding ethyl esters [40,41,100,102,103,104,105].

Without the above anchors, data fusion will only classify process states. It cannot show why the state changes. For example, a VOC model can indicate late fermentation, but it cannot determine whether the ethyl hexanoate level is low due to a lack of ethanol supply, insufficient hexanoic acid, weak esterification activity or excessive hydrolysis. Therefore, the two should be combined to design online monitoring and offline verification.

### 6.5. Data Fusion Should Be Interpretable

Artificial intelligence (AI) and big-data methods should be used as tools for soft sensing and pattern recognition, not as independent conclusions. Recent Baijiu studies have used multi-dimensional data fusion and interpretable machine learning for fermentation-stage recognition [42,106,107]. Other work has applied electronic nose and multi-omics data to characterize volatile and non-volatile changes in Baijiu [106]. These studies show that multi-source signals can improve process discrimination.

Data fusion can be employed to obtain some unobserved function information in real time, such as the concentration of living microorganisms, precursor conversion rates and ester trajectory dynamics. The model’s input may be temperature, moisture, O_2_, CO_2_, NIR spectra and VOC fingerprints. The output should be an exact biochemical endpoint, not all-weather quality standards. Model interpretability can be used to identify the signals that support a prediction, and controllable variables should be used for enterprises to avoid a black-box classification approach. The monitoring strategy should also match this functional-verification logic. Table 4 summarizes the main online and offline methods that can be combined to distinguish process-state changes from true microbial and enzymatic functional maintenance.

## 7. Boundary Conditions for Stress-Tolerant Functional Units in Gradient Solid-State Fermentation

### 7.1. Research Progress and Remaining Boundary

Existing studies have shown three levels of progress. First, spatially resolved and Daqu-centered studies have described heterogeneity in fermented grains, workshops, Daqu types, process rounds and starter microbiota [44,46,52,108]. Second, studies on active microbes, environmental sources, microbial succession, substrate effects and core interactions have shown that fermentation function is shaped by local process conditions rather than by taxonomy alone [25,29,30,61]. Third, biofilm, *Bacillus*, acid regulation, yeast fortification, *Pichia*-related ecology and fortified Daqu studies indicate that functional regulation is possible but is often strain-, layer- or process-dependent [75,91,92,96]. Pit-mud, vessel-age, regional Daqu, filamentous fungi, hydrolases and starter-comparison studies further show that boundary materials and enzyme sources change precursor supply and flavor trajectories [20,60,62,73]. These results support functional intervention, but they do not yet define the full operating boundary of stress-tolerant ester-producing units.

The unresolved problem is quantitative. A functional unit should not be described only by genus name or final ester increase. It should be described by stress parameters, reaction parameters and persistence parameters. Stress parameters include temperature, moisture, acidity, ethanol and oxygen limitation. Reaction parameters include ethanol formation, organic acid conversion, ester-related enzyme activity and target ester trajectory. Persistence parameters include viable abundance, repeated-cycle stability and layer consistency.

### 7.2. Gradient Stress Defines the Functional Window

The final output of future studies should be a functional window. This window should define the range in which a microbial unit maintains acid–alcohol–ester conversion. Reported values such as 10 g/L acetic acid tolerance, 12 g/L acetic acid tolerance, 50 °C heat response, 29.16% fortified pit-mud abundance, and layer-dependent ethyl hexanoate increases of 15.9–27.2% show that quantitative description is possible [32,79,81,84,85]. However, these values are isolated unless they are measured together with position, stage, moisture, gas state and ester output.

Therefore, the gradient reactor should not only ask whether a functional unit works. It should determine where the function fails. Loss of viable cells, reduced ethanol production, excessive acid accumulation, decreased esterification activity or unstable ester ratios can all indicate that the functional boundary has been crossed.

### 7.3. Evidence Standard

Future verification should require agreement among three types of data: gradient-resolved process variables, biological activity and chemical output. Process variables define the stress condition. Biological activity shows whether the selected microorganisms remain active. Chemical output shows whether acid–alcohol–ester conversion is maintained, and should be supported by yeast-derived ester evidence, volatile-compound profiling, trace-component analysis, aged-aroma comparison and cross-system metabolomics when needed [74,104,107]. Only when these three levels match can a functional unit be considered valid for engineering use.

This section therefore sets the boundary of the review. The focus is not the whole Baijiu microbiome, and not a general intelligent brewing system. The focus is whether stress-tolerant microbial units can maintain ester-producing metabolism under measurable solid-state gradients.

## 8. Conclusions

Baijiu solid-state fermentation is not only the spontaneous succession of microorganisms, but also a gradient-selected functional system. Temperature, moisture, pH, O_2_ limitation, ethanol, substrate availability and mass transfer jointly affect the activity of functional microorganisms and the transformation of acid–alcohol–ester metabolism. Final flavor quality therefore cannot be explained only by the presence of dominant microorganisms or by the endpoint concentration of single esters. The central question is whether stress-tolerant ester-producing functional units can maintain precursor transformation and ester formation under heterogeneous solid-state stress.

In this review, the functional unit is regarded as the basic object for understanding ester formation. Yeasts, lactic acid bacteria, *Bacillus*, filamentous fungi and anaerobic acid-producing bacteria should not be analyzed separately only according to taxonomic abundance. Their contribution should be judged by whether they remain active in a defined layer, stage and stress condition, and whether they can support acid supply, alcohol supply, esterification and ester retention in the same local process. This view shifts the focus from community description to functional continuity.

Gradient solid-state bioreactors are proposed here as a conceptual bridge between laboratory screening and traditional pit fermentation. They would not replace pit fermentation; instead, they would reproduce selected gradients and help define functional boundaries for microbial units. With the addition of online monitoring, the GC-MS, GC-IMS, NIR, qPCR, metagenomics, metabolomics and enzyme assay components in this system will offer more in-depth data on functional maintenance.

Future studies should identify which stress-tolerant functional units maintain the acid–alcohol–ester balance across repeated fermentation cycles and determine the gradient conditions at which that function fails. Such evidence could support more stable Baijiu fermentation and targeted ester-profile regulation while preserving the ecological characteristics of traditional solid-state production.

## Figures and Tables

**Table 1:** Research progress and bottlenecks of key gradients in Baijiu solid-state fermentation.

Gradient	Progress Line	Key Parameters	Main Bottleneck	Failure Boundary	Ref.
Temperature	Fermentation observation → spatial/temporal sampling → model-based mapping	Layer temperature, temperature difference (ΔT), heating rate, time above threshold	Heat is local and transient	Cells survive, but ester conversion drops	[6,52,53,64,65,66,67,68,69]
Moisture	Initial water control → layer moisture tracking	Moisture, water activity, migration rate	Sampling disturbs solid matrix	Enzyme diffusion and cell activity decouple	[6,7]
Acidity/pH	Total acid → acid species → precursor balance	pH and lactic, acetic, butyric, and hexanoic acids	Total acid hides pathway difference	Acid accumulates without ester formation	[6,13,60,61,62]
Ethanol	Yield index → stress and precursor index	Ethanol level, formation rate, acid/ethanol ratio	Product and inhibitor overlap	Ethanol rises, but ester ratio worsens	[50,55,61,62]
Oxygen/redox	Aerobic view → microaerobic/anaerobic boundary	O_2_, CO_2_, oxidation–reduction potential (ORP), pressure drop	Deep-bed gas state is hard to define	Respiration signal cannot prove ester function	[8,34,35,38,39]
Interface	Pit mud/Huangshui correlation → boundary module	Contact area, time, redox, feedback volume	Boundary effects are hard to isolate	Interface improves acids but not target esters	[56,57,58,59,63]

**Table 2:** Quantitative evidence for stress-tolerant ester-producing functional units.

Candidate Member or Unit	Reported Stress Parameter	Functional Readout	Evidence Meaning	Ref.
*Schizosaccharomyces pombe*	10 g/L acetic acid tolerance	Acetic acid in fermented grains decreased from 9.62 to 6.55 g/kg	Links acid tolerance with acid regulation in a fermentation matrix	[32]
*Pichia kudriavzevii*	Heat-response study under 50 °C stress	Transcriptomic and metabolomic evidence of heat response	Candidate for high-temperature Daqu or upper-layer heat stress	[79,80]
*Pichia kudriavzevii* from Huangshui	12 g/L acetic acid tolerance	Acetic acid tolerance mechanism and survival evidence	Candidate for acidic liquid-feedback regions	[81]
*Saccharomyces cerevisiae* YS219	Organic acid tolerance	Changed microbial community and flavor metabolites	Ethanol producer with possible ester-related contribution	[24,25]
Yeast–*Bacillus* co-culture	Co-culture or biofilm-associated state	Changed ester synthesis and flavor-related metabolism	Spatial persistence may affect ester formation.	[18]
Yeast–anaerobe co-culture	Anaerobic acid-production coupling	Enhanced ethyl hexanoate-related metabolism in simplified systems	Couples ethanol supply with C4–C6 acid formation	[82,83]
*Caproicibacterium lactatifermentans* in pit mud	29.16% abundance after fortified pit-mud intervention	Promoted fatty acid and ethyl ester formation during short-term use	Shows short-term pit-mud functional enhancement	[84]
Fortified Daqu functional unit	Top/middle/bottom layer-dependent response	Ethyl hexanoate increased by 15.9%, 15.5% and 27.2%; ethyl acetate decreased by 5.4%, 11.1% and 26.6%.	Demonstrates that functional regulation is position-dependent	[85]

**Table 3:** Solid-state bioreactor development, parameter comparison and failure risks.

Reactor Type	Development Role	Key Parameters	Main Limitation	Baijiu-Oriented Use
Traditional pit	Real ecosystem reference	Layer depth, pit age, interface, cycle	Uncontrolled and hard to repeat	Source of gradient targets
Small SSF model	Low-cost mechanistic test	Substrate mass, moisture, inoculum, stage	Scale and heat transfer are weak	Pre-screen functional units
Tray reactor	Simple solid-state operation	Layer thickness, surface area, humidity	Weak deep-bed anaerobic gradient	Daqu-like aerobic process reference
Packed-bed	Static bed with gas control	Bed height, gas flow, pressure drop (ΔP), O_2_/CO_2_	Heat and moisture gradients accumulate	Core platform for gradient testing
Zymotis-type	Heat-transfer improved static bed	Plate spacing, coolant T, internal ΔT	Too much control may flatten gradient	Separate heat stress from other factors
Multi-layer reactor	Position-resolved validation	Layer thickness, sampling depth, transfer path	Layer interaction is difficult to quantify	Define functional window by layer
Rotating drum	Homogenized solid-state process	Rotation speed, fill ratio, mixing interval	Destroys spatial structure	Useful as non-gradient control

**Table 4:** Monitoring technology progress, bottlenecks and validation requirements.

Method	Progress Line	Useful Parameter	Main Bottleneck	Required Anchor
GC-MS/GC-IMS	Endpoint detection → compound trajectory	Key esters, acids, alcohols, VOCs	Offline and sampling-dependent	Same-point microbial data
qPCR/PMA-qPCR	Abundance → viable target tracking	Copy number, viable signal, primer specificity	Activity is still indirect	Metabolites and enzyme activity
Gas sensors	Manual gas check → continuous O_2_/CO_2_	O_2_, CO_2_, response time, drift	Respiration is non-specific	Ethanol, acids and target microbes
NIR	Lab calibration → rapid process screening	Spectral interval, root-mean-square error of prediction (RMSEP), calibration set	Model transfer is weak	Wet-chemistry calibration
Raman	Molecular fingerprint → cell/metabolite signal	Update time, signal/noise, peak assignment	Solid matrix interference	Chromatography and qPCR
E-nose/VOC	Aroma fingerprint → batch warning	Sensor array, drift, classification accuracy	Cannot identify causal compounds	GC-MS/GC-IMS
Multimodal AI	Single sensor → data fusion prediction	Feature set, validation batch, external test	Overfitting and poor interpretability	Mechanistic and independent validation

## Data Availability

No new data were created or analyzed in this study. Data sharing is not applicable to this article.
